# Association of Thyroid Dysfunction With Cognitive Function: An Individual Participant Data Analysis

**DOI:** 10.1001/jamainternmed.2021.5078

**Published:** 2021-09-07

**Authors:** Nicolien A. van Vliet, Diana van Heemst, Osvaldo P. Almeida, Bjørn O. Åsvold, Carole E. Aubert, Jong Bin Bae, Linda E. Barnes, Douglas C. Bauer, Gerard J. Blauw, Carol Brayne, Anne R. Cappola, Graziano Ceresini, Hannie C. Comijs, Jean-Francois Dartigues, Jean-Marie Degryse, Robin P. F. Dullaart, Marlise E. A. van Eersel, Wendy P. J. den Elzen, Luigi Ferrucci, Howard A. Fink, Leon Flicker, Hans J. Grabe, Ji Won Han, Catherine Helmer, Martijn Huisman, M. Arfan Ikram, Misa Imaizumi, Renate T. de Jongh, J. Wouter Jukema, Ki Woong Kim, Lewis H. Kuller, Oscar L. Lopez, Simon P. Mooijaart, Jae Hoon Moon, Elisavet Moutzouri, Matthias Nauck, Jim Parle, Robin P. Peeters, Mary H. Samuels, Carsten O. Schmidt, Ulf Schminke, P. Eline Slagboom, Eystein Stordal, Bert Vaes, Henry Völzke, Rudi G. J. Westendorp, Michiko Yamada, Bu B. Yeap, Nicolas Rodondi, Jacobijn Gussekloo, Stella Trompet

**Affiliations:** 1 Department of Internal Medicine, Section of Gerontology and Geriatrics, Leiden University Medical Center, Leiden, the Netherlands; 2 Medical School, University of Western Australia, Perth, Western Australia, Australia; 3 Western Australian Centre for Health and Ageing, University of Western Australia, Perth, Western Australia, Australia; 4 K.G. Jebsen Center for Genetic Epidemiology, Department of Public Health and Nursing, NTNU, Norwegian University of Science and Technology, Trondheim, Norway; 5 Department of Endocrinology, Clinic of Medicine, St Olav’s Hospital, Trondheim University Hospital, Trondheim, Norway; 6 HUNT Research Center, Department of Public Health and Nursing, NTNU, Norwegian University of Science and Technology, Levanger, Norway; 7 Department of General Internal Medicine, Inselspital, Bern University Hospital, University of Bern, Bern, Switzerland; 8 Institute of Primary Health Care (BIHAM), University of Bern, Bern, Switzerland; 9 Center for Clinical Management Research, Veterans Affairs Ann Arbor Healthcare System, Ann Arbor, Michigan; 10 Institute for Healthcare Policy and Innovation, University of Michigan, Ann Arbor; 11 Department of Neuropsychiatry, Seoul National University Bundang Hospital, Seongnam, South Korea; 12 Department of Public Health and Primary Care, Cambridge Institute of Public Health, University of Cambridge, Cambridge, United Kingdom; 13 Division of General Internal Medicine, School of Medicine, University of California, San Francisco; 14 Division of Endocrinology, Diabetes, and Metabolism, Department of Medicine, Perelman School of Medicine at the University of Pennsylvania, Philadelphia; 15 Department of Medicine and Surgery, University of Parma, Unit of Internal Medicine and Oncological Endocrinology, University Hospital of Parma, Parma, Italy; 16 Department of Psychiatry, Amsterdam Public Health research institute, Amsterdam UMC, Vrije Universiteit Amsterdam, Amsterdam, the Netherlands; 17 GGZ inGeest Specialized Mental Health Care, Research and Innovation, Amsterdam, the Netherlands; 18 UMR 1219, Bordeaux Population Health Research Center, Inserm, University of Bordeaux, Bordeaux, France; 19 Department of Public Health and Primary Care, Katholieke Universiteit Leuven, Leuven, Belgium; 20 Institute of Health and Society, Université catholique de Louvain, Brussels, Belgium; 21 Division of Endocrinology, Department of Internal Medicine, University of Groningen, University Medical Center Groningen, Groningen, the Netherlands; 22 University Center for Geriatric Medicine, University of Groningen, University Medical Center Groningen, Groningen, the Netherlands; 23 Department of Clinical Chemistry and Laboratory Medicine, Leiden University Medical Center, Leiden, the Netherlands; 24 Atalmedial Diagnostics Centre, Amsterdam, the Netherlands; 25 Department of Clinical Chemistry, Amsterdam UMC, Amsterdam, the Netherlands; 26 Longitudinal Studies Section, Translational Gerontology Branch, Harbor Hospital, Baltimore, Maryland; 27 National Institute on Aging NIA-ASTRA Unit, Baltimore, Maryland; 28 Geriatric Research Education and Clinical Center, VA Healthcare System, Minneapolis, Minnesota; 29 Department of Medicine, University of Minnesota, Minneapolis; 30 Department of Psychiatry and Psychotherapy, University Medicine Greifswald, Greifswald, Germany; 31 German Center for Neurodegenerative Diseases (DZNE), Site Rostock/Greifswald, Germany; 32 Department of Epidemiology and Biostatistics, Amsterdam Public Health Research Institute, Amsterdam UMC, Vrije Universiteit Amsterdam, Amsterdam, the Netherlands; 33 Department of Sociology, VU University Amsterdam, Amsterdam, the Netherlands; 34 Department of Epidemiology, Erasmus MC, Rotterdam, the Netherlands; 35 Department of Clinical Studies, Radiation Effects Research Foundation, Hiroshima and Nagasaki, Japan; 36 Department of Internal Medicine and Endocrinology, Amsterdam UMC, Amsterdam, the Netherlands; 37 Department of Cardiology, Leiden University Medical Center, Leiden, the Netherlands; 38 Netherlands Heart Institute, Utrecht, the Netherlands; 39 Department of Brain and Cognitive Science, Seoul National University College of Natural Sciences, Seoul, South Korea; 40 Department of Psychiatry, Seoul National University, College of Medicine, Seoul, South Korea; 41 Department of Epidemiology, Graduate School of Public Health, University of Pittsburgh, Pittsburgh, Pennsylvania; 42 Department of Neurology, University of Pittsburgh School of Medicine, Pittsburgh, Pennsylvania; 43 Department of Internal Medicine, Seoul National University Bundang Hospital, Seongnam, South Korea; 44 Institute of Clinical Chemistry and Laboratory Medicine, University Medicine Greifswald, Greifswald, Germany; 45 DZHK (German Centre for Cardiovascular Research), partner site, Greifswald, Germany; 46 Institute of Clinical Sciences, University of Birmingham, Birmingham, United Kingdom; 47 Department of Internal Medicine, Erasmus MC, Rotterdam, the Netherlands; 48 Academic Center for Thyroid Diseases, Erasmus MC, Rotterdam, the Netherlands; 49 Division of Endocrinology, Diabetes and Clinical Nutrition, Department of Medicine, Oregon Health & Science University, Portland; 50 Department of Radiology, University Medicine Greifswald, Greifswald, Germany; 51 Department of Neurology, University Medicine Greifswald, Greifswald, Germany; 52 Department of Biomedical Data Sciences, Section of Molecular Epidemiology, Leiden University Medical Center, Leiden, the Netherlands; 53 Max Planck Institute for Biology of Ageing, Cologne, Germany; 54 Namsos Hospital, Nord-Trøndelag Hospital Trust, Namsos, Norway; 55 Department of Mental Health, NTNU, Norwegian University of Science and Technology, Trondheim, Norway; 56 Institute for Community Medicine, University Medicine Greifswald, Greifswald, Germany; 57 Department of Public Health, Section of Epidemiology, Center for Healthy Aging, University of Copenhagen, Copenhagen, Denmark; 58 Department of Endocrinology and Diabetes, Fiona Stanley Hospital, Western Australia, Australia; 59 Department of Public Health and Primary Care, Leiden University Medical Center, Leiden, the Netherlands

## Abstract

**Importance:**

In clinical guidelines, overt and subclinical thyroid dysfunction are mentioned as causal and treatable factors for cognitive decline. However, the scientific literature on these associations shows inconsistent findings.

**Objective:**

To assess cross-sectional and longitudinal associations of baseline thyroid dysfunction with cognitive function and dementia.

**Design, Setting, and Participants:**

This multicohort individual participant data analysis assessed 114 267 person-years (median, 1.7-11.3 years) of follow-up for cognitive function and 525 222 person-years (median, 3.8-15.3 years) for dementia between 1989 and 2017. Analyses on cognitive function included 21 cohorts comprising 38 144 participants. Analyses on dementia included eight cohorts with a total of 2033 cases with dementia and 44 573 controls. Data analysis was performed from December 2016 to January 2021.

**Exposures:**

Thyroid function was classified as overt hyperthyroidism, subclinical hyperthyroidism, euthyroidism, subclinical hypothyroidism, and overt hypothyroidism based on uniform thyrotropin cutoff values and study-specific free thyroxine values.

**Main Outcomes and Measures:**

The primary outcome was global cognitive function, mostly measured using the Mini-Mental State Examination. Executive function, memory, and dementia were secondary outcomes. Analyses were first performed at study level using multivariable linear regression and multivariable Cox regression, respectively. The studies were combined with restricted maximum likelihood meta-analysis. To overcome the use of different scales, results were transformed to standardized mean differences. For incident dementia, hazard ratios were calculated.

**Results:**

Among 74 565 total participants, 66 567 (89.3%) participants had normal thyroid function, 577 (0.8%) had overt hyperthyroidism, 2557 (3.4%) had subclinical hyperthyroidism, 4167 (5.6%) had subclinical hypothyroidism, and 697 (0.9%) had overt hypothyroidism. The study-specific median age at baseline varied from 57 to 93 years; 42 847 (57.5%) participants were women. Thyroid dysfunction was not associated with global cognitive function; the largest differences were observed between overt hypothyroidism and euthyroidism—cross-sectionally (−0.06 standardized mean difference in score; 95% CI, –0.20 to 0.08; *P* = .40) and longitudinally (0.11 standardized mean difference higher decline per year; 95% CI, –0.01 to 0.23; *P* = .09). No consistent associations were observed between thyroid dysfunction and executive function, memory, or risk of dementia.

**Conclusions and Relevance:**

In this individual participant data analysis of more than 74 000 adults, subclinical hypothyroidism and hyperthyroidism were not associated with cognitive function, cognitive decline, or incident dementia. No rigorous conclusions can be drawn regarding the role of overt thyroid dysfunction in risk of dementia. These findings do not support the practice of screening for subclinical thyroid dysfunction in the context of cognitive decline in older adults as recommended in current guidelines.

## Key Points

**Question:**

Is thyroid dysfunction associated with cognitive decline?

**Findings:**

In this individual participant data analysis of 23 cohorts including 74 565 participants with cognitive function and/or dementia measurements, subclinical thyroid dysfunction was not associated with global cognitive function at baseline (standardized mean difference, −0.02 for subclinical hyperthyroidism and 0.05 for subclinical hypothyroidism) or annual decline (standardized mean difference, −0.02 for subclinical hyperthyroidism and −0.00 for subclinical hypothyroidism).

**Meaning:**

These findings do not support the need for screening for subclinical thyroid dysfunction for prevention of cognitive decline or dementia.

## Teaser

This individual participant data analysis assesses the cross-sectional and longitudinal associations of baseline thyroid dysfunction with cognitive function and dementia.

## Introduction

Thyroid dysfunction is considered a potentially reversible cause of cognitive decline; hence, thyroid function screening tests are described in guidelines as an essential component of the workup for the diagnosis of dementia.^1,2,3^ Thyroid dysfunction is frequently observed in individuals with suspected dementia.^4^ However, the outcomes of treatment of overt hypothyroidism and hyperthyroidism and subclinical hyperthyroidism on cognitive function are not fully clarified.^5,6,7^ For subclinical hypothyroidism, 4 of 5 recent randomized clinical trials and a meta-analysis on levothyroxine treatment did not find evidence for an improvement in cognitive function.^8,9,10,11,12,13^ Moreover, meta-analyses of observational studies have yielded inconsistent results on associations of subclinical and overt thyroid dysfunction with cognitive impairment and risk of dementia.^14,15,16,17^ An individual participant data analysis of cohort studies might help clarify the conflicting results of previous studies, as it allows for uniform definitions of thyroid dysfunction and it can assess the differential associations by age groups, sex, and thyroid medication in subgroup analyses.^18^ In the present study, we investigated cross-sectional and longitudinal associations of thyroid dysfunction with cognitive function and dementia in an individual participant data analysis of multiple cohorts.

## Methods

### Study Population

We first approached the coordinating center of the Thyroid Studies Collaboration, a collaborative project of 25 existing longitudinal studies with information on thyroid status.^18^ The Medical Ethics Committee of the Leiden University Medical Center waived the need for review owing to the retrospective nature of the study using only previously collected data; no individuals underwent interventions for the present study. Each participant gave informed consent to the original study they participated in, which was oral or written depending on the original study design and legislation at the time of data collection. All 15 Thyroid Studies Collaboration cohorts that had collected data on cognitive function or dementia joined the project. The study designs for all cohorts participating in the current study have been described previously in more detail.^19,20,21,22,23,24,25,26,27,28,29,30,31,32,33^ We approached 14 additional cohorts that were extracted from 4 recent meta-analyses on subclinical thyroid dysfunction and cognitive function or dementia.^14,15,16,17^ Six of these cohorts consented to collaborating and sharing data.^34,35,36,37,38,39^ Lastly, we included publicly available data of the National Health and Nutrition Examination Survey waves of 1999 to 2002 and 2011 to 2012, which simultaneously collected thyroid and cognitive function among many other parameters.^40^

### Thyroid Function

Thyroid dysfunction was determined biochemically by measurements of thyrotropin and free thyroxine (FT_4_) concentrations in all cohorts. Cohort-specific cutoff values were used for FT_4_ levels (eTable 1 in the Supplement). In accordance with previous projects in the Thyroid Studies Collaboration, participants were classified as euthyroid if thyrotropin level was 0.45 to 4.49 mIU/L.^18^ Overt hyperthyroidism was defined as a thyrotropin level less than 0.45 mIU/L and FT_4_ level above the reference range. Subclinical hyperthyroidism was defined as a thyrotropin level less than 0.45 mIU/L and FT_4_ levels within the reference range, or only as thyrotropin level less than 0.45 mIU/L in absence of an FT_4_ measurement (n = 896 among 10 cohorts) because overt hyperthyroidism is rare.^41^ A combination of thyrotropin level of 4.50 to 20 mIU/L and FT_4_ levels within the reference range was defined as subclinical hypothyroidism. Individuals who had missing FT_4_ measurements with mildly elevated thyrotropin levels (4.50-20 mIU/L) were considered subclinically hypothyroid (n = 523 among 8 cohorts) because chances of overt hypothyroidism in this patient category are low.^41^ A thyrotropin level of 20 mIU/L or greater or thyrotropin level of 4.50 mIU/L or greater combined with FT_4_ levels below the reference range was defined as overt hypothyroidism.

### Cognitive Function

The primary outcome was global cognitive function measured by Mini-Mental State Examination (MMSE), Modified Mini-Mental State (3MS), or Severe Cognitive Impairment Rating Scale.^42,43,44^ A difference of 1 point in MMSE score is considered the minimal clinically important difference in individuals without dementia.^45^ Executive function and memory were secondary outcomes. For executive function, various tests were used: Digit Symbol Substitution Test, Trail Making Test B, Letter Digit Substitution Test (LDST), Executive Interview 15, and Ruff Figural Fluency Test.^46,47,48,49,50^ The minimal clinically important difference for executive function was defined as a difference of 4 points in LDST.^51^ Memory was measured using either Rey’s Auditory Verbal Learning Test (also referred to as Word Learning Test or Verbal Learning Test), Digit Span Test, or Visual Association Test.^52,53,54,55^ No minimal clinically important difference for memory tests was found in the literature.

### Dementia

Depending on the study design, dementia was diagnosed either in a clinical setting or at a research center. The diagnosis was, at least in part, based on clinical presentation. Studies in which dementia diagnosis was based only on a cutoff point for the MMSE were excluded from this analysis because cognitive function tests are insufficient to diagnose dementia.^56^ Prevalence of dementia at baseline was available for 11 cohorts; 431 participants had a diagnosis of dementia at baseline, but only 78 of them were classified as noneuthyroid. Owing to the small number of participants with thyroid dysfunction at baseline, no cross-sectional analyses for dementia were performed.

### Statistical Analyses

We used a 2-stage individual participant data analysis approach, which accommodates uniform definitions and analyses for each cohort while keeping complexity to a minimum.^18,57^ The first stage consisted of study-level analysis of thyroid dysfunction and cognitive function or dementia conducted on the original data sets with participant-level data. In the second stage, the effect estimates from the first stage were pooled using a random-effects model based on restricted maximum likelihood. Heterogeneity across studies was quantified using the *I*^2^ statistic: less than 40% was considered low heterogeneity; 40% to 75%, moderate heterogeneity; and greater than 75%, high heterogeneity.

For both the cross-sectional and longitudinal analyses between thyroid dysfunction and cognitive function, we used multivariable linear regression models. To facilitate combination of different scales, the results were transformed to standardized mean differences. In the prospective analysis of cognitive decline, we calculated the difference between the last available measurement of cognitive function and baseline cognitive function. The difference was divided by the follow-up time in years to obtain an annual decline, irrespective of duration of follow-up. The annual decline was subsequently standardized, resulting in a standardized mean difference in annual change in cognitive function allowing comparison of changes over time.

The risk of developing dementia during follow-up was assessed using Cox regression models. In these analyses, participants with dementia at baseline were excluded. For studies without precise registration of the date of dementia diagnosis, it was assumed that dementia developed halfway between the registration date and the last date that absence of dementia was ascertained.

Thyroid dysfunction (overt hyperthyroidism, subclinical hyperthyroidism, subclinical hypothyroidism, and overt hypothyroidism) was included as a categorical variable with the euthyroid group serving as reference. All analyses were adjusted for age and sex. The longitudinal analyses of cognitive decline were adjusted for baseline cognitive function. Prespecified subgroup analyses were performed by stratification and interaction analysis for sex and for age younger or older than 75 years. Additional analyses were performed with adjustment for educational attainment, though this variable was not available in all cohorts. In sensitivity analyses, participants with missing FT_4_ measurements in the subclinical hyperthyroid and subclinical hypothyroid groups were excluded, as were those who used antithyroid medication or thyroid hormone replacement therapy at baseline. Furthermore, we assessed robustness of the associations by pooling the estimates using fixed-effect models and by excluding studies with strata of fewer than 10 participants. To assess whether effects were dependent on degree of disruption of thyrotropin, analyses were repeated with thyrotropin categories of less than 0.10 mIU/L, 0.10 to 0.44 mIU/L, 4.5 to 6.9 mIU/L, 7.0 to 10 mIU/L, and greater than 10 mIU/L, in which participants with thyrotropin between 0.45 and 4.49 mIU/L served as reference. Lastly, instead of using biochemical cutoff points, thyrotropin and FT_4_ were analyzed continuously across the full range with cognitive function. Thyrotropin was transformed using the natural logarithm; for both natural log-transformed thyrotropin and FT_4_, models were constructed per standard deviation. Continuous models were performed minimally adjusted by age and sex and with additional adjustment for educational attainment. For sensitivity purposes, the analyses were also conducted excluding the participants who used antithyroid medication or thyroid hormone replacement therapy at baseline. Cohorts with greater than 10% missing measurements for FT_4_ were excluded for the continuous analyses on FT_4_. All *P* values were 2-tailed; statistical significance was set at *P* < .05.

Study-level analyses were performed using SPSS Statistics, version 25 (IBM). Effect estimates were pooled and summarized in forest plots using R, version 3.6.1 and metafor package (R Foundation for Statistical Computing).^58^ This study followed the Strengthening the Reporting of Observational Studies in Epidemiology (STROBE) reporting guideline.

## Results

### Population Characteristics

Individual participant data on thyroid function and cognitive function and/or dementia were provided by 23 cohorts comprising 74 565 participants. At baseline, 66 567 (89.3%) participants were biochemically classified as euthyroid, 577 (0.8%) as overtly hyperthyroid, 2557 (3.4%) as subclinically hyperthyroid, 4167 (5.6%) as subclinically hypothyroid, and 697 (0.9%) as overtly hypothyroid (eTable 1 in the Supplement). The study-specific median age at baseline varied from 57 to 93 years; 42 847 (57.5%) participants were women.

A total of 38 144 participants from 21 cohorts provided data on a measure of cognitive function (Table). The median age varied from 58 to 93 years, and 18 089 (47.4%) participants were women. Follow-up for cognitive decline was available for 14 cohorts, with a median follow-up duration varying from 1.7 to 11.3 years, accumulating 114 267 person-years.

**Table.** Baseline Characteristics of the 38 144 Participants With Cognitive Function Measurements in Included Studies

Source	Location	Population description	Baseline, y	No.	Age, median (range), y	No. (%)				Cognitive function		Follow-up duration,^c^ median (range), y
						Men	Women	Euthyroid^a^	Thyroid medication users	Scales	Score,^b^ mean (SD)	
**Europe**												
BELFRAIL cohort study	Belgium	Adults aged ≥80 y	2008-2009	523	84 (80-102)	193 (36.9)	330 (63.1)	453 (86.6)	50 (9.6)^d^	MMSE	26 (4.0)	1.7 (0.5-2.3)
BETS	England	Community-dwelling adults aged ≥65 y	2002-2004	5845	72 (65-98)	2873 (49.2)	2972 (50.8)	5266 (90.1)	0 (0)	MMSE	28 (2.2)	0
CFAS	England and Wales	Adults aged ≥64 y	1991-1992	1015	73 (64-94)	497 (49.0)	518 (51.0)	906 (89.3)	NA	MMSE	28 (2.0)	2.0 (1.9-2.6)
InCHIANTI Study	Italy	Community-dwelling adults	1998-2000	1187	71 (21-102)	521 (43.9)	666 (56.1)	1044 (88.0)	33 (2.8)	MMSE	25 (4.8)	9.0 (2.8-10.0)
LASA	The Netherlands	Adults aged ≥65 y	1995-1997	1266	75 (65-89)	616 (48.7)	650 (51.3)	1093 (86.3)	26 (2.1)	MMSE, WLT	27 (3.1)	9.9 (2.3-20.8)
Leiden 85-plus Study	The Netherlands	Adults aged 85 y	1997-1999	557	85	188 (33.8)	369 (66.2)	456 (81.9)	20 (3.6)	MMSE, LDST, VLT	24 (6.3)	5.0 (1.0-5.0)
LLS	The Netherlands	Long-lived siblings	2002-2005	776	93 (89-103)	308 (39.7)	468 (60.3)	652 (84.0)	NA	MMSE	24 (5.1)	0
Paquid study	France	Community-dwelling adults aged ≥65 y	1989-1990	407	75 (66-94)	173 (42.5)	234 (57.5)	359 (88.2)	6 (1.5)^d^	MMSE, DSST, VLT	26 (3.5)	11.3 (1.5-27.0)
PREVEND Study	The Netherlands	Adults	2003-2006	864	58 (35-82)	493 (57.1)	371 (42.9)	777 (89.9)	NA	RFFT, VAT	64 (25.0)	5.2 (0.8-7.8)
PROSPER	The Netherlands, Ireland, Scotland	Older community-dwelling adults at high cardiovascular risk	1998-1999	5775	75 (69-83)	2791 (48.3)	2984 (51.7)	5063 (87.7)	256 (4.4)	MMSE, LDST, WLT	28 (1.5)	3.3 (0.8-4.0)
Rotterdam Study	The Netherlands	Adults aged ≥55 y	1989-1992	1875	69 (55-93)	720 (38.4)	1155 (61.6)	1611 (85.9)	46 (2.5)^d^	MMSE	28 (1.7)	10.8 (1.5-21.7)
SHIP	Germany	Adults	2002-2006	1329	69 (60-88)	682 (51.3)	647 (48.7)	1008 (75.8)	190 (14.3)	MMSE	28 (3.2)	5.6 (4.3-8.8)
**North America**												
CHS	US	Community-dwelling adults with Medicare eligibility	1994-1998	3991	74 (64-98)	1635 (41.0)	2356 (59.0)	3253 (81.5)	401 (10.0)^d^	3MS, DSST	90 (9.9)	5.9 (0.9-7.0)
HABC Study	US	Community-dwelling adults aged 70-79 y with Medicare eligibility	1999-2000	2488	75 (71-82)	1208 (48.6)	1280 (51.4)	2076 (83.4)	251 (10.1)	3MS, EXIT 15	90 (8.9)	8.0 (2.0-13.0)
MMC	Mexico	Geriatric outpatients with and without dementia	2004	156	79 (58-98)	49 (31.4)	107 (68.6)	109 (69.9)	12 (7.7)	MMSE	15 (6.5)	0
MrOS Study	US	Community-dwelling men aged ≥65 y	2000-2002	1600	73 (65-99)	1600 (100)	0	1409 (88.1)	122 (7.6)^d^	3MS, TMT	93 (6.4)	4.6 (3.5-5.9)
NHANES 1999-2002	US	Adults	1999-2002	853	70 (60-85)	416 (48.8)	437 (51.2)	751 (88.0)	91 (10.7)	DSST	42 (18.3)	0
NHANES 2011-2012	US	Adults	2011-2012	434	68 (60-80)	220 (50.7)	214 (49.3)	405 (93.3)	57 (13.1)	DSST, WLT	45 (17.6)	0
**Australia**												
HIMS	Australia	Men aged ≥65 y	2001-2004	3168	76 (71-89)	3168 (100)	0	2897 (91.4)	112 (3.5)	MMSE	28 (1.3)	0
**Asia**												
KLOSCAD	Republic of Korea	Adults aged ≥60 y	2010-2017	3854	70 (61-109)	1702 (44.2)	2152 (55.8)	3476 (90.2)	NA	SCIRS, TMT, DST	29 (1.7)	3.7 (0.8-7.3)
KLOSHA	Republic of Korea	Adults aged ≥65 y	2010-2012	181	75 (70-96)	2 (1.1)	179 (98.9)	154 (85.1)	NA	MMSE, TMT, DST	24 (3.9)	0
Overall		21 Cohorts	1989-2017	38 144	74 (21-109)	20 055 (52.6)	18 089 (47.4)	33 218 (87.1)	1673 (5.3)			5.4 (0.5-27.0)

Abbreviations: BETS, Birmingham Elderly Thyroid Study; CFAS, Cognitive Function and Aging Study; CHS, Cardiovascular Health Study; DSST, Digit Symbol Substitution Test; DST, Digit Span Test; EXIT 15, 15-item Executive Interview; HABC, Health, Aging and Body Composition; HIMS, Health in Men Study; InCHIANTI, Invecchiare in Chianti Study; KLOSCAD, Korean Longitudinal Study on Cognitive Aging and Dementia; KLOSHA, Korean Longitudinal Study on Health and Aging; LASA, Longitudinal Aging Study Amsterdam; LDST, Letter Digit Substitution Test; LLS, Leiden Longevity Study; MMC, Mexican Memory Clinic; MMSE, Mini-Mental State Examination; MrOS, Osteoporotic Fractures in Men Study; NA, not available; NHANES, National Health and Nutrition Examination Survey; Paquid, Personnes-Agées QUID; PREVEND, Prevention of Renal and Vascular End-stage Disease; PROSPER, Prospective Study of Pravastatin in the Elderly at Risk; RFFT, Ruff Figural Fluency Test; SCIRS; Severe Cognitive Impairment Rating Scale; SHIP, Study of Health in Pomerania; 3MS, Modified Mini-Mental State Examination; TMT, Trail Making Test; VAT, Visual Association Test; VLT, Verbal Learning Test; WLT, Word Learning Test.

^a^ We used a common definition for biochemical euthyroidism of thyrotropin level of 0.45 to 4.49 mIU/L, resulting in different numbers from previous reports.

^b^ Test scores are shown for global cognitive function tests. If no global cognitive function test scores were provided, executive function test scores are shown.

^c^ Follow-up in years for participants who had a follow-up measurement for cognitive function.

^d^ Data on baseline medication use (thyroid replacement therapy, antithyroid drugs) were unavailable for 2 participants of the BELFRAIL Study, 3 participants of the CHS, 64 participants of the MrOS Study, 12 participants of the PAQUID Study, 1 participant of the Rotterdam Study.

Eight cohorts provided follow-up for dementia incidence on 46 606 participants (eTable 2 in the Supplement). Among these participants, 28 820 (61.8%) were women, and the median age at baseline was between 57 and 85 years. During follow-up, 2033 (4.4%) cases of incident dementia were identified. Median follow-up duration ranged from 3.8 to 15.3 years, accumulating 525 222 person-years.

### Thyroid Dysfunction and Global Cognitive Function

**Figure 1.**
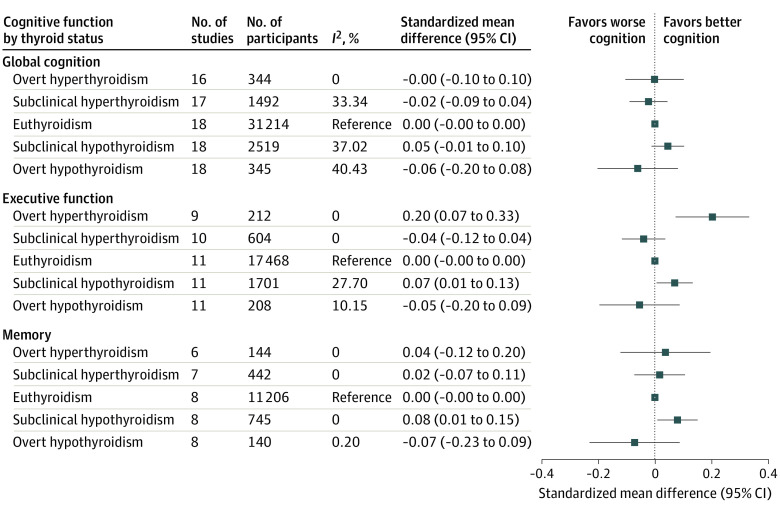
Cross-sectional Association Between Thyroid Dysfunction and Cognitive Function Test Scores Standardized mean differences were adjusted for age and sex. Error bars indicate 95% CIs.

**Figure 2.**
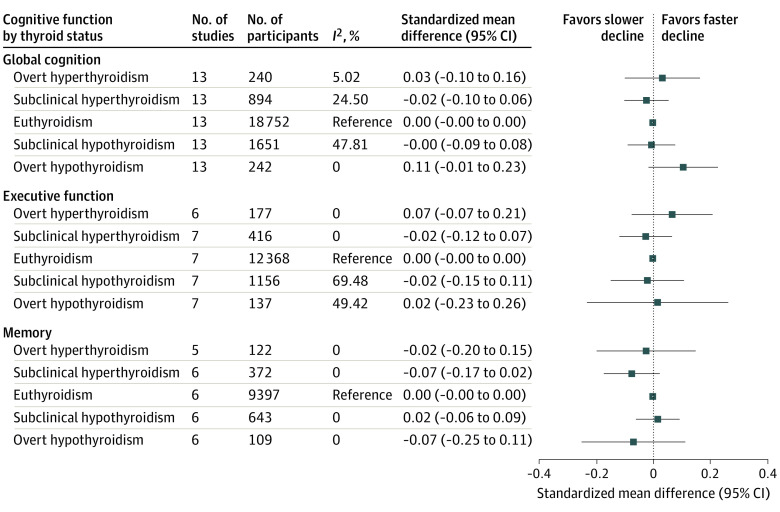
Longitudinal Association Between Thyroid Dysfunction and Cognitive Function Test Scores Standardized mean differences were adjusted for age, sex, and baseline cognitive function. Error bars indicate 95% CIs.

Cross-sectionally, thyroid dysfunction was not associated with global cognitive function among 18 cohorts (Figure 1; eFigure 1 in the Supplement). The largest observed difference was −0.06 standardized mean difference (95% CI, −0.20 to 0.08; *P* = .40) global cognitive function for overt hypothyroidism compared with euthyroidism, which could be interpreted as an approximately 0.1-point lower MMSE score based on the SD for the 2 largest cohorts included. No statistically significant association was observed between thyroid dysfunction at baseline and annual change in global cognitive function during follow-up among 13 cohorts (Figure 2). Participants with overt hypothyroidism had 0.11 standardized mean difference (95% CI, −0.01 to 0.23; *P* = .09) higher decline per year in global cognitive function than participants who were euthyroid, which translates to approximately 0.1 point on the MMSE scale faster decline per year based on the SD in the largest cohort for this analysis. Additional adjustment for educational attainment did not materially change the results (eFigure 2 in the Supplement). Stratification by age and sex did not show any differential effects for global cognitive function (eTable 3 in the Supplement). No statistically significant associations were found when individuals were categorized by severity of thyrotropin abnormality (eFigure 3 in the Supplement). Reanalyzing the data with a fixed-effects model or without strata with fewer than 10 participants did not yield different results (eTable 4 in the Supplement). Leaving out participants with missing FT_4_ measurements or those using antithyroid medication or thyroid hormone replacement therapy at baseline also did not change the results. A positive association was found between continuous thyrotropin and global cognition only when thyroid supplementation and antithyroid medication users were excluded (0.028 higher standardized mean difference per SD; 95% CI, 0.003 to 0.053; *P* = .03; eTable 5 in the Supplement). No association between continuous FT_4_ levels and global cognitive function was found. Heterogeneity across studies was low for the cross-sectional main analyses (*I*^2^ = 0%-40%), while heterogeneity was low to moderate for the longitudinal and sensitivity analyses (*I*^2^ = 0%-70%).

### Thyroid Dysfunction and Executive Function and Memory

No negative association was observed cross-sectionally between thyroid dysfunction and executive function or memory among 11 and 8 cohorts, respectively (Figure 1; eFigures 4 and 5 in the Supplement). Participants with overt hyperthyroidism had 0.20 standardized mean difference (95% CI, 0.07 to 0.33; *P* = .002) higher executive function score compared with participants who were euthyroid; transformed, this would account for 1.6 more correct substitutions within 60 seconds for the LDST based on the largest cohort in this analysis. In both executive function and memory, participants with subclinical hypothyroidism performed better than participants who were euthyroid (executive function: 0.07 standardized mean difference; 95% CI, 0.01 to 0.13; *P* = .03; memory: 0.08 standardized mean difference; 95% CI, 0.01 to 0.15; *P* = .03). Longitudinally, no association was found between thyroid dysfunction at baseline and decline in executive function among 7 cohorts or memory among 6 cohorts; all differences were smaller than 0.1 standardized mean difference (Figure 2). Additional adjustment for educational attainment did not materially change the results (eFigure 2 in the Supplement). No statistically significant interaction with sex or age was present (*P* > .05 for all; supporting data in eTable 3 in the Supplement). Using a fixed-effects model or excluding strata with fewer than 10 participants did not change the results for executive function or memory (eTable 4 in the Supplement). The association of subclinical hypothyroidism and better executive function was attenuated when participants with missing FT_4_ measurements were left out, while the association with memory was unchanged. The positive association between overt hyperthyroidism and executive function disappeared when participants using thyroid medication were removed. No association was found when individuals were categorized by severity of thyrotropin abnormality or when thyrotropin level was analyzed continuously (eFigure 3 and eTable 5 in the Supplement). Continuous analysis of FT_4_ levels showed a positive association with executive function (0.019 higher standardized mean difference per SD; 95% CI, 0.002 to 0.036; *P* = .03), which was attenuated when participants using thyroid medication were left out. Heterogeneity across studies was low for the cross-sectional main analyses (*I*^2^ = 0%-40%), while heterogeneity was low to moderate for the longitudinal analyses (*I*^2^ = 0%-70%) and up to high heterogeneity in the sensitivity analyses (*I*^2 ^≤ 73%).

### Thyroid Dysfunction and Dementia

**Figure 3.**
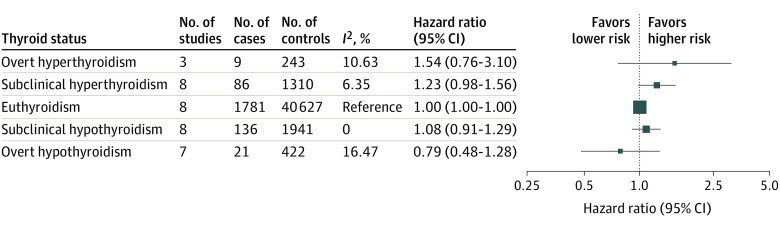
Longitudinal Association Between Thyroid Dysfunction and Incident Dementia Hazard ratios were adjusted for age and sex. Error bars indicate 95% CIs.

Cross-sectional analysis of thyroid dysfunction and dementia were unfeasible owing to few participants who were not euthyroid with dementia at baseline (78 participants among 11 cohorts). In longitudinal analyses among 12 cohorts, no association was found between thyroid dysfunction and incident dementia (Figure 3; eFigure 6 in the Supplement). The hazard ratio of dementia ranged from 1.54 (95% CI, 0.76 to 3.10) for overt hyperthyroidism to 0.79 (95% CI, 0.48 to 1.28) for overt hypothyroidism. Continuous analysis of thyrotropin and FT_4_ levels also did not provide evidence for an association; hazard ratio, 0.96 per SD increase of natural log-transformed thyrotropin (95% CI, 0.91 to 1.02; *P* = .16); hazard ratio, 1.05 per SD increase of FT_4_ (95% CI, 0.98 to 1.13; *P* = .16). Heterogeneity between studies was low (*I*^2^ = 0%-40%).

## Discussion

In this individual participant data analysis of 74 565 participants from 23 cohorts, there was no association between subclinical thyroid dysfunction and cognitive function, cognitive decline, or the onset of dementia. Owing to uncertainty of the results for overt hypothyroidism and hyperthyroidism, no rigorous conclusions can be drawn regarding the association between overt thyroid dysfunction and cognitive decline and dementia.

While prior study-level meta-analyses also reported no association between subclinical hypothyroidism and cognitive function, cognitive decline, or dementia, they were limited by heterogeneity in definitions of thyroid dysfunction and choices of covariates in the statistical models.^14,15,16,17^ Because we performed an individual participant data analysis, we could standardize definitions of thyroid function categories and of cognitive function and cognitive decline and standardize the statistical models. By addressing these limitations and reaching the same results, the present study provides the strongest observational evidence to date suggesting that subclinical hypothyroidism is not associated with cognitive function or cognitive decline.

Various studies and 2 meta-analyses did show an association between subclinical or overt hyperthyroidism or low thyrotropin level within the reference range and a higher risk of dementia.^14,17,20,26,59,60,61^ Although our findings for subclinical and overt hyperthyroidism and dementia did not reach statistical significance, they are directionally consistent with the literature. Despite combining 8 cohorts comprising more than 45 000 participants, the number of individuals with subclinical and overt hyperthyroidism and the number of individuals who developed dementia during follow-up are limited. Therefore, we cannot exclude a higher risk of dementia in individuals with hyperthyroidism. In addition, individuals with overt hyperthyroidism had a slightly higher rate of cognitive decline, though not statistically significant. Considering the existing literature and the other results in the present study, the observed cross-sectional association between overt hyperthyroidism and better executive function was most likely a chance finding. Moreover, the observed difference in executive function was less than half the minimal clinically important difference, making it a clinically insignificant finding regardless of the *P* value.

Higher vulnerability among subgroups has been proposed; younger adults and women might be more susceptible to cognitive dysregulation associated with thyroid dysfunction.^16,62^ Moreover, cognitive decline might only be present in individuals with more extreme values of thyrotropin,^21,63^ or variation in FT_4_ instead of thyrotropin levels could be associated with dementia risk.^22^ In the present multicohort study, we did not observe differential associations for participants younger and older than 75 years or for men and women, nor any association with variation in FT_4_ level or more extreme values of thyrotropin. Therefore, subgroup associations reported in prior studies might not be generalizable outside the original cohorts.

As mentioned before, all but 1 randomized clinical trial on levothyroxine treatment for subclinical hypothyroidism also did not provide evidence for improvement of cognitive function.^8,9,10,11,12^ Moreover, both undertreatment and overtreatment with levothyroxine are common, estimated at 27% and 14%, respectively.^64^ Overtreatment is associated with increased risk of atrial fibrillation and atherosclerosis^65,66^ and, via cerebrovascular damage, might be associated with increased risk of cognitive decline. Therefore, screening for subclinical thyroid dysfunction in older adults to prevent cognitive impairment and dementia does not appear to be effective.

The current individual participant data analysis has several strengths. The use of individual participant data from cohorts from all over the globe enhances generalization while allowing standardized definitions and relevant subgroup analyses. All but 5 of the included studies had a median age of 70 years or older, which is essential but often not the case in research concerning outcomes that are most relevant for older adults.^67^ The present study approached cognition comprehensively; we assessed multiple domains of cognitive function, cross-sectionally and longitudinally, and incidence of dementia.

### Limitations

Some limitations need to be acknowledged. Thyroid function categorization was based on biochemical characteristics. For 20% to 30% of the participants who were categorized as subclinical hypothyroid or hyperthyroid, we could not confirm subclinical thyroid dysfunction owing to the absence of FT_4_ measurement. This may have led to some misclassification, yet sensitivity analyses excluding those participants with missing FT_4_ data yielded similar results. We could not include educational attainment in our main analysis because 5 out of 18 cohorts did not collect these data. Even though the sensitivity analyses with adjustment for educational attainment yielded similar results as the main analysis, education is a possible confounder that could not be accounted for. For most cohorts, only 1 measurement of thyroid function was available, which is why only baseline thyroid function was used in the present individual participant data analysis. This study could therefore not capture any changes in cognitive function that might occur at the transition of one thyroid status to another. Moreover, for the vast majority of study participants, a maximum of 2 measurements of cognitive function was available, which precluded advanced modeling of change over time including nonlinear trajectories. In addition, the interpretation of longitudinal studies of cognitive function can be complicated by practice effects.^68^ Standardization of change over time might not fully alleviate this; hence, residual practice effects may still be present. Furthermore, because dementia is clinically difficult to diagnose, some misclassification could have occurred, which may have led to an underestimation of the association. In addition, the number of incident dementia cases in the included cohort studies was low; we therefore cannot rule out a clinically relevant association between thyroid dysfunction and risk of dementia. The heterogeneity between studies may have been increased by the use of different cognitive function tests, different durations of follow-up, differences in age and sex distribution, different lifestyles across continents, and different inclusion criteria. As heterogeneity was expected a priori, we performed all meta-analyses with random effects. Nonetheless, results for fixed-effects meta-analyses were not materially different. The observed heterogeneity was larger in the longitudinal analyses heterogeneity (*I*^2^ = 0%-70%) than in the cross-sectional analyses (*I*^2^ = 0%-40%), likely owing to the additional variation of follow-up duration. We hypothesize that the minor differences in *I*^2^ estimates between different cross-sectional analyses are attributable to differences in sample size per exposure. Because individuals with thyroid disease generally receive medical treatment, we cannot address the question of whether long-term untreated hyperthyroidism or hypothyroidism is associated with cognitive function and dementia risk. Moreover, these results only apply to objectifiable cognitive decline, which is not synonymous with the more subjective cognitive complaints.

## Conclusions

In this individual participant data analysis combining the individual participant data of 74 565 participants from 23 cohorts, subclinical thyroid dysfunction was not associated with cognitive function, cognitive decline, or risk of dementia. Hence, it is unlikely that treatment for otherwise undetected subclinical thyroid dysfunction would improve cognitive function. Moreover, the chance of overtreatment is considerable, which increases the risk of atrial fibrillation, atherosclerosis, and cerebral infarction and thereby might increase the risk of cognitive decline. Whether treatment of overt hypothyroidism or hyperthyroidism is associated with cognitive decline and risk of dementia remains uncertain. Existing clinical guidelines that prescribe screening of subclinical thyroid dysfunction for prevention of cognitive decline or dementia should therefore be revisited.

## Notes

Supplement. **eTable 1.** Reference ranges for free thyroxine and distribution of biochemical thyroid status at baseline for 23 included cohorts **eTable 2.** Baseline Characteristics of the 46,606 Participants Included for Analyses on Incident Dementia **eTable 3.** Cross-sectional associations between Thyroid Dysfunction and Cognitive Function Test Scores stratified by Age and Sex **eTable 4.** Sensitivity analyses of cross-sectional associations between Thyroid Dysfunction and Cognitive Function Test Scores **eTable 5**. Association between full range TSH and fT4 and Cognitive Function Test Scores **eFigure 1.** Cross-sectional association between Thyroid Dysfunction and Global Cognitive Function **eFigure 2.** Cross-sectional association between Thyroid Dysfunction and Cognitive Function Test Scores additionally adjusted for education **eFigure 3.** Cross-sectional association between categorized TSH and Cognitive Function Test Scores **eFigure 4.** Cross-sectional association between Thyroid Dysfunction and Executive Function **eFigure 5.** Cross-sectional association between Thyroid Dysfunction and Memory **eFigure 6.** Longitudinal Association between Thyroid Dysfunction and Incident Dementia
