# Supplementary material for: Association of Thyroid Dysfunction With Cognitive Function: An Individual Participant Data Analysis
Source: JAMA Intern Med. 2021 Sep 7;181(11):1–12. doi: 10.1001/jamainternmed.2021.5078 (PMC8424529; doi:10.1001/jamainternmed.2021.5078)
Supplement: Supplement. — eTable 1. Reference ranges for free thyroxine and distribution of biochemical thyroid status at baseline for 23 included cohorts eTable 2. Baseline Characteristics of the 46,606 Participants Included for Analyses on Incident Dementia eTable 3. Cross-sectional associations between Thyroid Dysfunction and Cognitive Function Test Scores stratified by Age and Sex eTable 4. Sensitivity analyses of cross-sectional associations between Thyroid Dysfunction and Cognitive Function Test Scores eTable 5. Association between full range TSH and fT4 and Cognitive Function Test Scores eFigure 1. Cross-sectional association between Thyroid Dysfunction and Global Cognitive Function eFigure 2. Cross-sectional association between Thyroid Dysfunction and Cognitive Function Test Scores additionally adjusted for education eFigure 3. Cross-sectional association between categorized TSH and Cognitive Function Test Scores eFigure 4. Cross-sectional association between Thyroid Dysfunction and Executive Function eFigure 5. Cross-sectional association between Thyroid Dysfunction and Memory eFigure 6. Longitudinal Association between Thyroid Dysfunction and Incident Dementia [file jamainternmed-e215078-s001.pdf]

Supplemental Online Content

van Vliet NA, van Heemst D, Almeida OP, et al; for the Thyroid Studies Collaboration. Association of thyroid dysfunction with cognitive function: an individual participant data analysis. *JAMA Intern Med*. Published online September 7, 2021.
doi:10.1001/jamainternmed.2021.5078

eTable 1. Reference ranges for free thyroxine and distribution of biochemical thyroid status at baseline for 23 included cohorts

eTable 2. Baseline Characteristics of the 46,606 Participants Included for Analyses on Incident Dementia

eTable 3. Cross-sectional associations between Thyroid Dysfunction and Cognitive Function Test Scores stratified by Age and Sex

eTable 4. Sensitivity analyses of cross-sectional associations between Thyroid Dysfunction and Cognitive Function Test Scores

eTable 5. Association between full range TSH and fT4 and Cognitive Function Test Scores

eFigure 1. Cross-sectional association between Thyroid Dysfunction and Global Cognitive Function

eFigure 2. Cross-sectional association between Thyroid Dysfunction and Cognitive Function Test Scores additionally adjusted for education

eFigure 3. Cross-sectional association between categorized TSH and Cognitive Function Test Scores

eFigure 4. Cross-sectional association between Thyroid Dysfunction and Executive Function

eFigure 5. Cross-sectional association between Thyroid Dysfunction and Memory

eFigure 6. Longitudinal Association between Thyroid Dysfunction and Incident Dementia

This supplemental material has been provided by the authors to give readers additional information about their work.

© 2021 van Vliet NA et al. *JAMA Internal Medicine.*

eTable 1. Reference ranges for free thyroxine and distribution of biochemical thyroid status at baseline for 23 included cohorts

Study name	Reference range fT4	Overt hyperthyroidism, No. (%)	Subclinical hyperthyroidism, No. (%)	Euthyroidism, No. (%)	Subclinical hypothyroidism, No. (%)	Overt hypothyroidism, No. (%)
BELFRAIL	0.9 to 1.8 ng/dL	2 (0.4)	53 (10.1)	453 (86.6)	4 (0.8)	11 (2.1)
BETS	9.0 to 20.0 pmol/L	21 (0.4)	219 (3.7)	5,266 (90.1)	311 (5.3)	28 (0.5)
CFAS	13.0 to 23.0 pmol/L	7 (0.7)	23 (2.3)	906 (89.3)	39 (3.8)	40 (3.9)
CHS	0.7 to 1.7 ng/dL	69 (1.7)	113 (2.8)	3,253 (81.5)	528 (13.2)	28 (0.7)
Health ABC	0.8 to 1.8 ng/dL	7 (0.3)	72 (2.9)	2,076 (83.4)	309 (12.4)	24 (1.0)
HIMS	10.0 to 23.0 pmol/L	12 (0.3)	37 (1.0)	3,239 (91.2)	251 (7.1)	12 (0.3)
HUNT	8.0 to 20.0 pmol/L	210 (0.6)	938 (2.8)	31,218 (92.0)	1,309 (3.9)	240 (0.7)
InCHIANTI Study	0.77 to 2.19 ng/dL	16 (1.3)	86 (7.2)	1,044 (88.0)	33 (2.8)	8 (0.7)
KLOSCAD	0.89 to 1.76 ng/dL	31 (0.7)	175 (3.9)	4,019 (89.3)	221 (4.9)	57 (1.3)
KLOSHA	0.89 to 1.76 ng/dL	0 (0.0)	0 (0.0)	154 (85.1)	18 (9.9)	9 (5.0)
LASA	11.0 to 22.0 pmol/L	11 (0.9)	82 (6.5)	1,093 (86.3)	71 (5.6)	9 (0.7)
Leiden 85-plus Study	13.0 to 23.0 pmol/L	3 (0.5)	23 (4.1)	456 (81.9)	35 (6.3)	40 (7.2)
LLS	10.0 to 24.0 pmol/L	5 (0.6)	53 (6.8)	652 (84.0)	59 (7.6)	7 (0.9)
Mexican Memory Clinic	12.0 to 23.0 pmol/L	0 (0.0)	5 (3.2)	109 (69.9)	36 (23.1)	6 (3.8)
MrOS	0.70 to 1.85 ng/dL	2 (0.1)	30 (1.9)	1,409 (88.1)	148 (9.3)	11 (0.7)
NHANES 1999-2002[a]	69.5 to 164.7 nmol/L	5 (0.6)	30 (3.5)	751 (88.0)	52 (6.1)	15 (1.8)
NHANES 2011-2012	0.6 to 1.6 ng/dL	2 (0.5)	10 (2.3)	405 (93.3)	15 (3.5)	2 (0.5)
PAQUID study	16.0 to 29.0 pmol/L	3 (0.7)	20 (4.9)	359 (88.2)	17 (4.2)	8 (2.0)
PREVEND study	9.14 to 23.81 pmol/L	1 (0.1)	30 (3.5)	777 (89.9)	52 (6.0)	4 (0.5)
PROSPER study	12.0 to 18.0 pmol/L	109 (1.9)	127 (2.2)	5,063 (87.7)	443 (7.7)	33 (0.6)
RERF	0.8 to 2.5 ng/dL	0 (0.0)	46 (3.1)	1,245 (84.6)	102 (6.9)	79 (5.4)
Rotterdam Study	11.07 to 24.97 pmol/L	13 (0.7)	120 (6.4)	1,612 (85.9)	108 (5.8)	24 (1.3)
SHIP	8.3 to 18.9 pmol/L	48 (3.6)	265 (19.9)	1,008 (75.8)	6 (0.5)	2 (0.2)
**Overall**		577 (0.8)	2,557 (3.4)	66,567 (89.3)	4,167 (5.6)	697 (0.9)

© 2021 van Vliet NA et al. *JAMA Internal Medicine.*

Abbreviated study names: BETS, Birmingham Elderly Thyroid Study; CFAS, Cognitive Function and Ageing Study; CHS, Cardiovascular Health Study; Health ABC, Health, Aging and Body Composition Study; HIMS, Health in Men Study; HUNT, Trøndelag Health Study; InCHIANTI, Invecchiare in Chianti Study; KLOSCAD, Korean Longitudinal Study on Cognitive Aging and Dementia; KLOSHA, Korean Longitudinal Study on Health and Aging; LASA, Longitudinal Aging Study Amsterdam; LLS, Leiden Longevity Study; MrOS, Osteoporotic Fractures in Men Study; NHANES, National Health and Nutrition Examination Survey; PAQUID study, Personnes-Agées QUID study; PREVEND, Prevention of Renal and Vascular End-stage Disease Study; PROSPER, Prospective Study of Pravastatin in the Elderly at Risk; RERF, Radiation Effects Research Foundation; SHIP, Study of Health in Pomerania.

[a]Only total thyroxine was available

© 2021 van Vliet NA et al. *JAMA Internal Medicine.*

eTable 2. Baseline Characteristics of the 46,606 Participants Included for Analyses on Incident Dementia

**Study (Location)**	**Population description**	**Baseline, y**	**No.**	**Age, Median (Range), y**	**Women, No. (%)**	**Euthyroid participants[a], No. (%)**	**Thyroid medication users[b], No. (%)**	**Cases with dementia, No. (%)**	**Follow up Duration[c], Median (Range), y**
Europe									
HUNT Study (Norway)	Adults	1995-1997	33,915	57 (19-99)	23,276 (68.6)	31,218 (92.0)	1,548 (4.6)	547 (1.6)	14.1 (0.1-15.3)
LASA (the Netherlands)	Adults aged ≥65 y	1995-1997	1,051	74 (65-88)	571 (54.3)	902 (85.8)	22 (2.1)	133 (12.7)	9.9 (1.3-16.5)
Leiden 85-plus Study (the Netherlands)	Adults aged 85 y	1997-1999	483	85	314 (65.0)	394 (81.6)	16 (3.3)	64 (13.3)	5.0 (0.5-5.0)
PAQUID study (France)	Community dwelling older adults ≥65 y	1989-1990	358	74 (66-94)	200 (55.9)	322 (89.9)	5 (1.4)[b]	119 (33.2)	9.0 (0.2-27.0)
Rotterdam Study (the Netherlands)	Adults aged ≥55 y	1989-1992	1,865	69 (55-93)	1,149 (61.6)	1,601 (85.8)	46 (2.5)[b]	350 (18.8)	15.3 (0.9-21.3)
Australia									
HIMS(Australia)	Men aged ≥65 y	2001-2004	3,549	76 (71-89)	0 (0)	3,237 (91.2)	123 (3.5)	479 (13.5)	11.4 (0.1-14.1)
Asia									
KLOSCAD (Republic of Korea)	Adults aged ≥60 y	2010-2017	3,913	69 (59-94)	2,228 (56.9)	3,489 (89.2)	NA	134 (3.4)	3.8 (0.3-7.5)
RERF (Japan)	Atomic bomb survivors	2000-2003	1,472	74 (56-97)	1,082 (73.5)	1,245 (84.6)	94 (6.4)[b]	207 (14.1)	8.0 (0.3-10.8)
**Overall**	8 cohorts	1989-2017	46,606	74 (19-99)	28,820 (61.8)	42,408 (91.0)	1,854 (4.3)	2,033 (4.4)	9.5 (0.1-27.0)

© 2021 van Vliet NA et al. *JAMA Internal Medicine.*

Abbreviated study names: HIMS, Health in Men Study; HUNT, Trøndelag Health Study; KLOSCAD, Korean Longitudinal Study on Cognitive Aging and Dementia; LASA, Longitudinal Aging Study Amsterdam; PAQUID study, Personnes-Agées QUID study; RERF Study, Radiation Effects Research Foundation.
NA, Data not available.
[a] We used a common definition for biochemical euthyroidism of thyroid-stimulating hormone 0.45-4.49 mU/L, resulting in different numbers from previous reports
[b] Data on baseline medication use (thyroid replacement therapy, antithyroid drugs) were unavailable for 12 participants of the PAQUID Study, 1 participant of the RERF Study, 1 participant of the Rotterdam Study.
[c] Follow up for incident cases was ended at date of diagnosis or midway between the last wave without and first wave with dementia.

© 2021 van Vliet NA et al. *JAMA Internal Medicine.*

eTable 3. Cross-sectional associations between Thyroid Dysfunction and Cognitive Function Test Scores stratified by Age and Sex

	Overt Hyperthyroidism	Subclinical Hyperthyroidism	Euthyroidism	Subclinical Hypothyroidism	Overt Hypothyroidism	No. with Overt Hyperthyroidism/ Subclinical Hyperthyroidism/ Euthyroidism/ Subclinical Hypothyroidism/ Overt Hypothyroidism
**Global cognitive function**						
All	-0.00 (-0.10;0.10)	-0.02 (-0.09;0.04)	0 (Ref)	0.05 (-0.01;0.10)	-0.06 (-0.20;0.08)	344/1492/31,214/2519/345
Sex						
Men	0.03 (-0.19;0.25)	-0.03 (-0.10;0.05)	0 (Ref)	0.03 (-0.05;0.12)	-0.02 (-0.18; 0.15)	95/587/16,933/1155/123
Women	-0.00 (-0.13;0.12)	-0.04 (-0.12;0.05)	0 (Ref)	0.08 (0.03;0.14)	-0.10 (-0.26; 0.07)	259/905/14,279/1364/222
Age, years						
<75	0.04 (-0.08;0.15)	-0.03 (-0.08;0.03)	0 (Ref)	0.04 (-0.02;0.10)	-0.05 (-0.18; 0.09)	192/793/17,074/1186/157
≥75	-0.05 (-0.22;0.13)	-0.01 (-0.11;0.10)	0 (Ref)	0.06 (-0.01;0.12)	-0.09 (-0.28; 0.10)	152/699/14,140/1333/188
**Executive function**						
All	0.20 (0.07;0.33)	-0.04 (-0.12;0.04)	0 (Ref)	0.07 (0.01;0.13)	-0.05 (-0.20; 0.09)	212/604/17,468/1701/208
Sex						
Men	-0.02 (-0.41;0.36)	-0.04 (-0.15;0.08)	0 (Ref)	0.05 (-0.03;0.13)	-0.05 (-0.24; 0.15)	45/215/9003/736/87
Women	0.26 (0.11;0.41)	-0.04 (-0.14;0.07)	0 (Ref)	0.10 (0.03;0.16)	-0.04 (-0.27; 0.19)	167/389/8463/965/121
Age, years						
<75	0.27 (0.10;0.45)	-0.02 (-0.12;0.07)	0 (Ref)	0.02 (-0.04;0.09)	0.03 (-0.16;0.21)	120/350/10086/844/97
≥75	0.07 (-0.16;0.30)	-0.06 (-0.18;0.06)	0 (Ref)	0.12 (0.05;0.19)	-0.15 (-0.36;0.06)	92/254/7382/857/111
**Memory**						
All	0.04 (-0.12;0.20)	0.02 (-0.07;0.11)	0 (Ref)	0.08 (0.01;0.15)	-0.07 (-0.23; 0.09)	144/442/11,206/745/140
Sex						
Men	-0.04 (-0.37;0.28)	0.04 (-0.11;0.20)	0 (Ref)	0.18 (0.04;0.31)	0.03 (-0.25;0.30)	34/156/5397/262/45
Women	0.06 (-0.12;0.25)	0.00 (-0.11;0.11)	0 (Ref)	0.03 (-0.07;0.12)	-0.12 (-0.33;0.09)	110/286/5807/483/95
Age, years						
<75	-0.01 (-0.22;0.20)	0.08 (-0.04;0.19)	0 (Ref)	0.04 (-0.05;0.14)	-0.04 (-0.27;0.19)	78/256/6599/413/64
≥75	0.09 (-0.16;0.33)	-0.07 (-0.21;0.07)	0 (Ref)	0.13 (0.02;0.24)	-0.08 (-0.32;0.17)	66/186/4607/332/76

© 2021 van Vliet NA et al. *JAMA Internal Medicine.*

eTable 4. Sensitivity analyses of cross-sectional associations between Thyroid Dysfunction and Cognitive Function Test Scores

	Global cognitive function		Executive function		Memory	
	**N cases/N controls**	**SMD (95% CI)**	**N cases/N controls**	**SMD (95% CI)**	**N cases/N controls**	**SMD (95% CI)**
**Overt Hyperthyroidism**						
Random-effects model	344/30951	-0.00 (-0.11; 0.10)	212/16989	0.20 (0.07; 0.33)	144/10674	0.04 (-0.12; 0.20)
Fixed-effects model	344/30951	-0.00 (-0.11; 0.10)	212/16989	0.20 (0.07; 0.33)	144/10674	0.04 (-0.12; 0.20)
Excluding strata <10 participants	315/24643	0.02 (-0.09; 0.13)	192/11275	0.23 (0.09; 0.36)	138/9291	0.02 (-0.14; 0.19)
Excluding participants using thyroid medication at baseline	165/25080	-0.09 (-0.32; 0.15)	79/11447	0.07 (-0.19; 0.33)	77/6397	-0.05 (-0.27; 0.17)
**Subclinical Hyperthyroidism**						
Random-effects model	1492/31060	-0.02 (-0.09; 0.05)	604/17348	-0.04 (-0.12; 0.04)	442/11054	0.02 (-0.07; 0.11)
Fixed-effects model	1492/31060	-0.03 (-0.08; 0.03)	604/17348	-0.04 (-0.12; 0.04)	442/11054	0.02 (-0.07; 0.11)
Excluding strata <10 participants	1487/30951	-0.02 (-0.09; 0.05)	604/17348	-0.04 (-0.12; 0.04)	442/11054	0.02 (-0.07; 0.11)
Excluding participants using thyroid medication at baseline	1018/25186	-0.08 (-0.18; 0.01)	264/12494	-0.08 (-0.20; 0.04)	216/6772	0.07 (-0.06; 0.21)
Excluding participants with missing free thyroxine	1148/31060	-0.03 (-0.11; 0.05)	494/17348	-0.03 (-0.12; 0.05)	353/11054	0.03 (-0.08; 0.13)
**Subclinical Hypothyroidism**						
Random-effects model	2519/31214	0.05 (-0.01; 0.10)	1701/17468	0.07 (0.01; 0.14)	745/11206	0.08 (0.01; 0.15)
Fixed-effects model	2519/31214	0.05 (0.01; 0.09)	1701/17468	0.08 (0.03; 0.12)	745/11206	0.08 (0.01; 0.15)

© 2021 van Vliet NA et al. *JAMA Internal Medicine.*

Excluding strata <10 participants	2509/29753	0.04 (-0.02; 0.10)	1701/17468	0.07 (0.01; 0.14)	745/11206	0.08 (0.01; 0.15)
Excluding participants using thyroid medication at baseline	2002/25186	0.04 (-0.02; 0.10)	1280/12494	0.09 (0.01; 0.16)	479/6772	0.08 (-0.01; 0.17)
Excluding participants with missing free thyroxine	1995/31214	0.01 (-0.06; 0.08)	1259/17468	0.03 (-0.03; 0.09)	515/11206	0.10 (0.02; 0.18)
**Overt Hypothyroidism**						
Random-effects model	345/31214	-0.06 (-0.20; 0.08)	208/17468	-0.05 (-0.20; 0.09)	140/11206	-0.07 (-0.23; 0.09)
Fixed-effects model	345/31214	-0.08 (-0.19; 0.02)	208/17468	-0.06 (-0.19; 0.07)	140/11206	-0.07 (-0.23; 0.09)
Excluding strata <10 participants	296/26797	-0.06 (-0.23; 0.11)	187/15831	-0.08 (-0.23; 0.07)	109/8601	-0.09 (-0.30; 0.12)
Excluding participants using thyroid medication at baseline	208/25186	-0.05 (-0.25; 0.15)	122/12494	-0.06 (-0.30; 0.17)	66/6772	-0.20 (-0.51; 0.10)

© 2021 van Vliet NA et al. *JAMA Internal Medicine.*

eTable 5. Association between full range TSH and fT4 and Cognitive Function Test Scores

	TSH			fT4		
	**N**	**Per SD of lnTSH**	**I^2**	**N**	**Per SD**	**I^2**
**Global cognition**						
Minimally adjusted	35,901	0.012 (-0.006; 0.030)	63.56%	22,514	0.006 (-0.007; 0.018)	0.00%
Adjusted for education	26,725	0.016 (-0.001; 0.033)	48.37%	14,845	0.002 (-0.014; 0.018)	7.94%
Excluding participants using thyroid medication	28,571	**0.028 (0.003; 0.053)**	70.49%	15,973	-0.000 (-0.021; 0.020)	25.97%
**Executive function**						
Minimally adjusted	20,192	0.018 (-0.002; 0.038)	45.48%	11,042	**0.019 (0.002; 0.036)**	0.00%
Adjusted for education	20,165	0.011 (-0.005; 0.027)	29.24%	11,018	0.015 (-0.001: 0.032)	0.00%
Excluding participants using thyroid medication	14,238	0.040 (-0.001; 0.081)	73.11%	5,681	0.003 (-0.028; 0.034)	4.44%
**Memory**						
Minimally adjusted	12,673	0.006 (-0.011; 0.023)	0.16%	5,598	0.012 (-0.013; 0.037)	0.00%
Adjusted for education	12,656	0.000 (-0.016; 0.016)	0.19%	5,581	0.014 (-0.009, 0.036)	0.00%
Excluding participants using thyroid medication	7,607	-0.012 (-0.047; 0.024)	29.67%	804	0.012 (-0.069; 0.094)	0.00%

For the continuous associations of fT4 with cognition cohorts with >10% missing data were excluded; Health ABC, LASA, Mexican Memory Clinic cohort, PAQUID, PROSPER, Rotterdam Study, SHIP. NHANES 1999-2002 was excluded because only total T4 was measured.
Data on educational attainment was not available for BETS, CFAS, LLS, MMC and SHIP.
Data on medication use was unavailable for CFAS, KLOSCAD, KLOSHA, LLS and PREVEND.

© 2021 van Vliet NA et al. *JAMA Internal Medicine.*

Study name	No. of Participants with Thyroid disorder	No. of Participants with Euthyroidism	Weight, %	Standardized Mean Difference (95% CI)
**Overt Hyperthyroidism**				
BELFRAIL	2	453	0.57	-1.44 [-2.81, -0.07]
BETS	21	5266	5.96	-0.02 [-0.44, 0.41]
CFAS	7	906	2.08	0.49 [-0.22, 1.21]
CHS	69	3253	19.99	0.08 [-0.15, 0.31]
Health ABC	7	2074	1.91	0.15 [-0.60, 0.90]
HIMS	10	2897	2.84	0.10 [-0.51, 0.72]
InCHIANTI	16	1044	6.75	-0.04 [-0.43, 0.36]
KLOSCAD	21	3459	6.37	0.07 [-0.34, 0.48]
LASA	11	1091	3.50	-0.06 [-0.61, 0.50]
Leiden 85-plus Study	3	456	0.82	-1.58 [-2.72, -0.43]
LLS	5	652	1.46	-0.85 [-1.71, 0.01]
MrOS	2	1408	0.59	-0.33 [-1.68, 1.02]
PAQUID	3	359	1.12	0.50 [-0.48, 1.47]
PROSPER	106	5014	29.58	-0.02 [-0.21, 0.17]
Rotterdam Study	13	1611	3.76	-0.33 [-0.86, 0.21]
SHIP	48	1008	12.71	0.13 [-0.16, 0.42]
Overall (I2=0.00%)	344	30951	100.00	-0.00 [-0.10, 0.10]
**Subclinical Hyperthyroidism**				
BELFRAIL	53	453	4.54	0.15 [-0.13, 0.42]
BETS	219	5266	11.06	-0.02 [-0.15, 0.12]
CFAS	23	906	2.49	0.35 [-0.05, 0.75]
CHS	113	3253	8.02	-0.02 [-0.20, 0.16]
Health ABC	72	2074	5.69	0.02 [-0.21, 0.26]
HIMS	30	2897	3.03	0.16 [-0.19, 0.52]
InCHIANTI	86	1044	7.72	-0.38 [-0.57, -0.19]
KLOSCAD	173	3459	10.24	0.01 [-0.14, 0.15]
LASA	82	1091	6.73	-0.00 [-0.21, 0.21]
Leiden 85-plus Study	23	456	2.29	0.04 [-0.38, 0.45]
LLS	53	652	4.64	-0.02 [-0.29, 0.25]
MMC	5	109	0.56	-0.40 [-1.29, 0.48]
MrOS	30	1408	3.06	0.03 [-0.32, 0.39]
PAQUID	20	359	2.45	-0.29 [-0.69, 0.12]
PROSPER	126	5014	8.44	-0.08 [-0.25, 0.09]
Rotterdam Study	119	1611	8.12	0.07 [-0.11, 0.25]
SHIP	265	1008	10.92	-0.03 [-0.16, 0.11]
Overall (I2=33.34%)	1492	31060	100.00	-0.02 [-0.09, 0.04]
**Subclinical Hypothyroidism**				
BELFRAIL	4	453	0.36	0.33 [-0.63, 1.29]
BETS	311	5266	10.75	0.01 [-0.11, 0.12]
CFAS	39	906	2.96	-0.00 [-0.31, 0.30]
CHS	528	3253	12.88	0.11 [0.02, 0.20]
Health ABC	309	2074	10.29	0.21 [0.09, 0.33]
HIMS	221	2897	9.03	-0.06 [-0.19, 0.08]
InCHIANTI	33	1044	3.45	0.12 [-0.17, 0.40]
KLOSCAD	136	3459	7.48	0.16 [0.00, 0.33]
KLOSHA	18	154	1.66	0.20 [-0.23, 0.63]
LASA	71	1091	4.84	-0.13 [-0.35, 0.10]
Leiden 85-plus Study	35	456	2.43	0.06 [-0.28, 0.41]
LLS	59	652	3.97	-0.05 [-0.31, 0.21]
MMC	36	109	2.27	0.17 [-0.19, 0.53]
MrOS	148	1408	7.27	-0.11 [-0.27, 0.06]
PAQUID	17	359	1.61	0.05 [-0.38, 0.49]
PROSPER	440	5014	12.20	0.03 [-0.07, 0.13]
Rotterdam Study	108	1611	6.06	-0.04 [-0.24, 0.15]
SHIP	6	1008	0.50	0.46 [-0.35, 1.27]
Overall (I2=37.02%)	2519	31214	100.00	0.05 [-0.01, 0.10]
**Overt Hypothyroidism**				
BELFRAIL	11	453	4.17	-0.20 [-0.79, 0.40]
BETS	28	5266	7.55	0.36 [-0.01, 0.73]
CFAS	40	906	8.81	-0.36 [-0.67, -0.05]
CHS	28	3253	7.67	0.13 [-0.23, 0.49]
Health ABC	24	2074	6.79	0.11 [-0.30, 0.51]
HIMS	10	2897	3.97	0.12 [-0.49, 0.74]
InCHIANTI	8	1044	4.52	0.01 [-0.55, 0.57]
KLOSCAD	47	3459	9.57	-0.18 [-0.46, 0.10]
KLOSHA	9	154	4.25	0.34 [-0.25, 0.93]
LASA	9	1091	3.94	-0.56 [-1.18, 0.05]
Leiden 85-plus Study	40	456	8.49	-0.13 [-0.45, 0.19]
LLS	7	652	3.11	-0.02 [-0.74, 0.70]
MMC	6	109	2.68	0.22 [-0.57, 1.01]
MrOS	11	1408	4.35	0.34 [-0.23, 0.92]
PAQUID	8	359	3.95	-0.35 [-0.96, 0.27]
PROSPER	33	5014	8.18	-0.52 [-0.86, -0.18]
Rotterdam Study	24	1611	7.04	-0.00 [-0.39, 0.39]
SHIP	2	1008	0.97	0.14 [-1.26, 1.54]
Overall (I2=40.43%)	345	31214	100.00	-0.06 [-0.20, 0.08]

RE Model

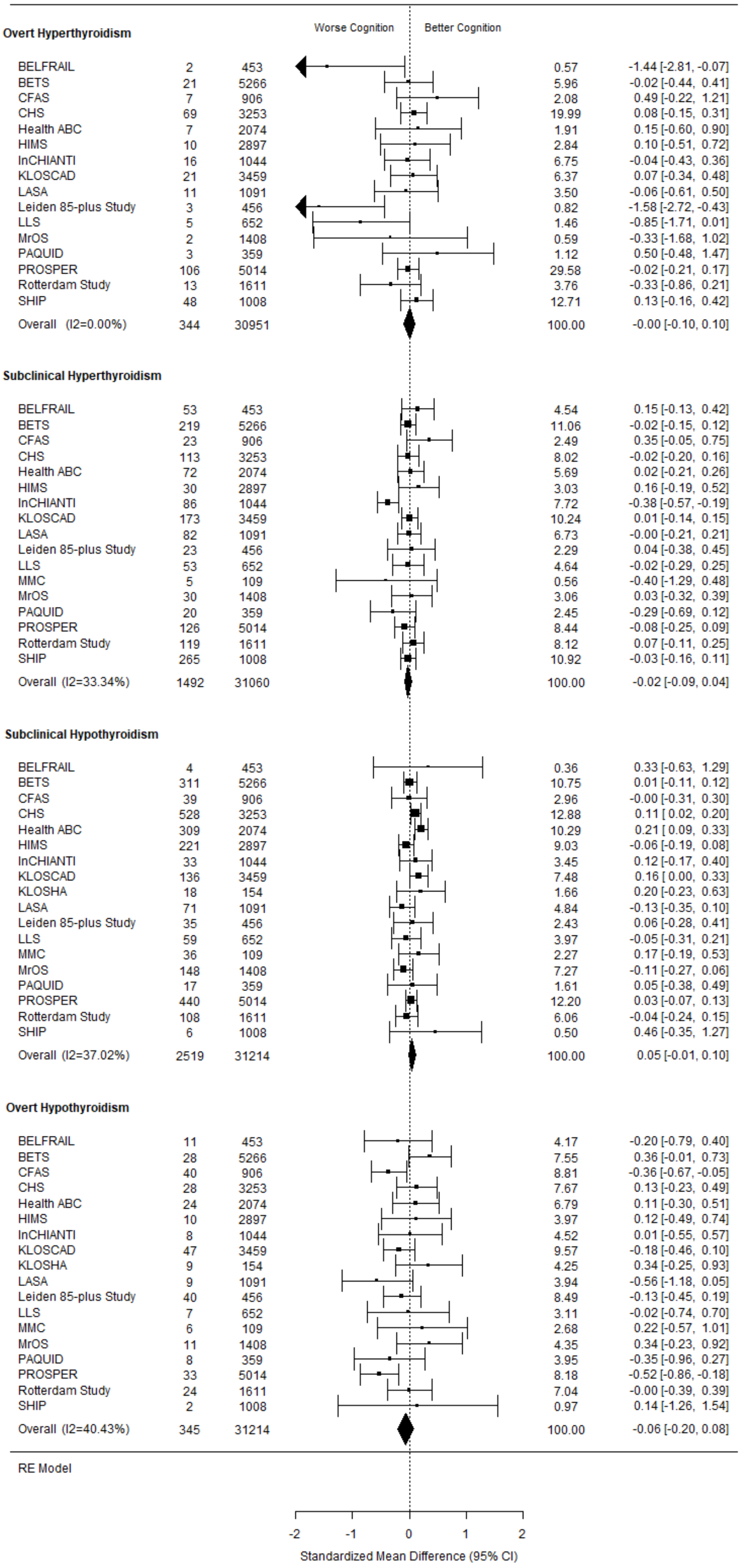

© 2021 van Vliet NA et al. *JAMA Internal Medicine.*

eFigure 1. Cross-sectional association between Thyroid Dysfunction and Global Cognitive Function

Standardized mean differences (SMDs) were adjusted for age and sex. Error bars indicate 95% confidence intervals. Abbreviated study names: BETS, Birmingham Elderly Thyroid Study; CFAS, Cognitive Function and Ageing Study; CHS, Cardiovascular Health Study; Health ABC, Health, Aging and Body Composition Study; HIMS, Health in Men Study; InCHIANTI, Invecchiare in Chianti Study; KLOSCAD, Korean Longitudinal Study on Cognitive Aging and Dementia; KLOSHA, Korean Longitudinal Study on Health and Aging; LASA, Longitudinal Aging Study Amsterdam; LLS, Leiden Longevity Study; MMC, Mexican Memory Clinic; MrOS, Osteoporotic Fractures in Men Study; PAQUID study, Personnes-Agées QUID study; PROSPER, Prospective Study of Pravastatin in the Elderly at Risk; SHIP, Study of Health in Pomerania.

© 2021 van Vliet NA et al. *JAMA Internal Medicine.*

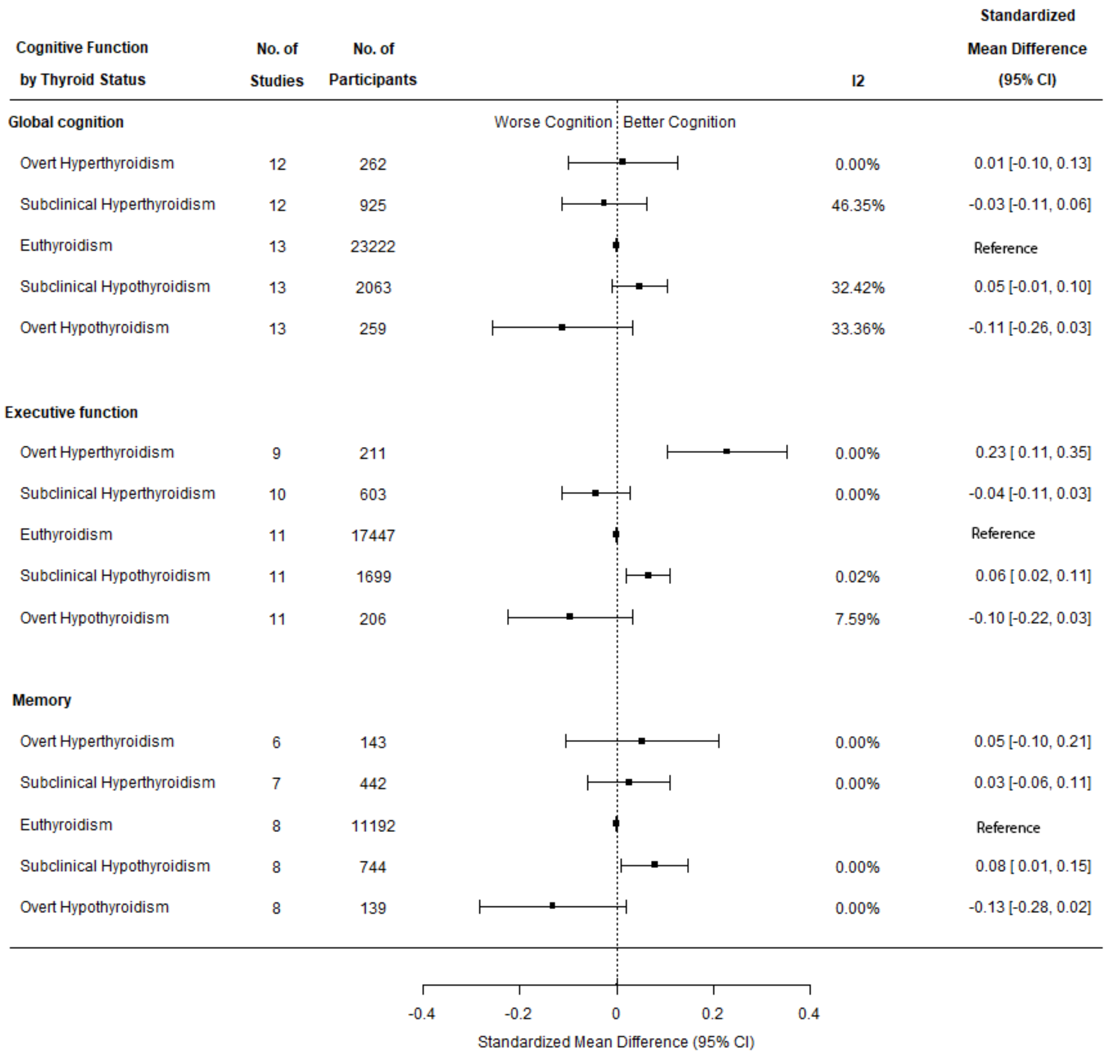

eFigure 2. Cross-sectional association between Thyroid Dysfunction and Cognitive Function Test Scores additionally adjusted for education

Standardized mean differences (SMDs) were adjusted for age, sex and educational attainment. Error bars indicate 95% confidence intervals.

© 2021 van Vliet NA et al. *JAMA Internal Medicine.*

Cognitive Function by TSH, mIU/L	No. of Studies	No. of Participants	I2	Standardized Mean Difference (95% CI)
**Global cognition**				
<0.10	15	516	0.00%	0.01 [-0.07, 0.10]
0.10-0.44	17	1312	47.55%	-0.04 [-0.12, 0.05]
0.45-4.49	18	31216		Reference
4.5-6.9	18	1842	30.86%	0.04 [-0.03, 0.10]
7.0-9.9	17	536	6.00%	0.03 [-0.06, 0.12]
>10	18	484	43.08%	0.02 [-0.11, 0.15]
**Executive function**				
<0.10	9	307	0.00%	0.07 [-0.04, 0.18]
0.10-0.44	10	508	41.19%	-0.02 [-0.14, 0.11]
0.45-4.49	11	17470		Reference
4.5-6.9	11	1247	50.70%	0.06 [-0.03, 0.15]
7.0-9.9	10	359	0.00%	0.04 [-0.06, 0.14]
>10	11	301	15.95%	0.07 [-0.06, 0.19]
**Memory**				
<0.10	6	198	0.00%	0.12 [-0.01, 0.26]
0.10-0.44	7	387	0.00%	-0.03 [-0.12, 0.07]
0.45-4.49	8	11206		Reference
4.5-6.9	8	566	0.00%	0.02 [-0.06, 0.10]
7.0-9.9	7	163	43.06%	0.08 [-0.17, 0.32]
>10	8	156	0.00%	0.09 [-0.06, 0.24]

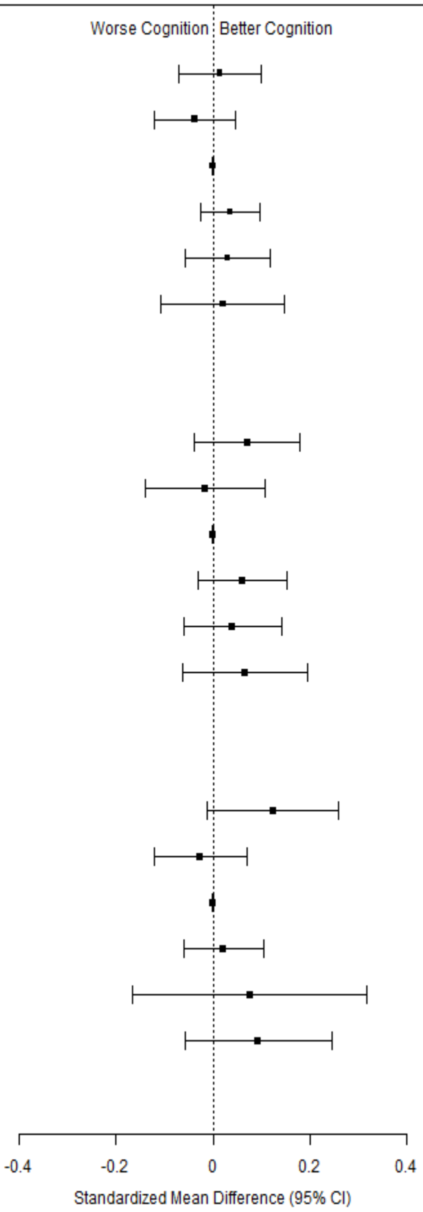

eFigure 3. Cross-sectional association between categorized TSH and Cognitive Function Test Scores

Standardized mean differences (SMDs) were adjusted for age and sex. Error bars indicate 95% confidence intervals.

© 2021 van Vliet NA et al. *JAMA Internal Medicine.*

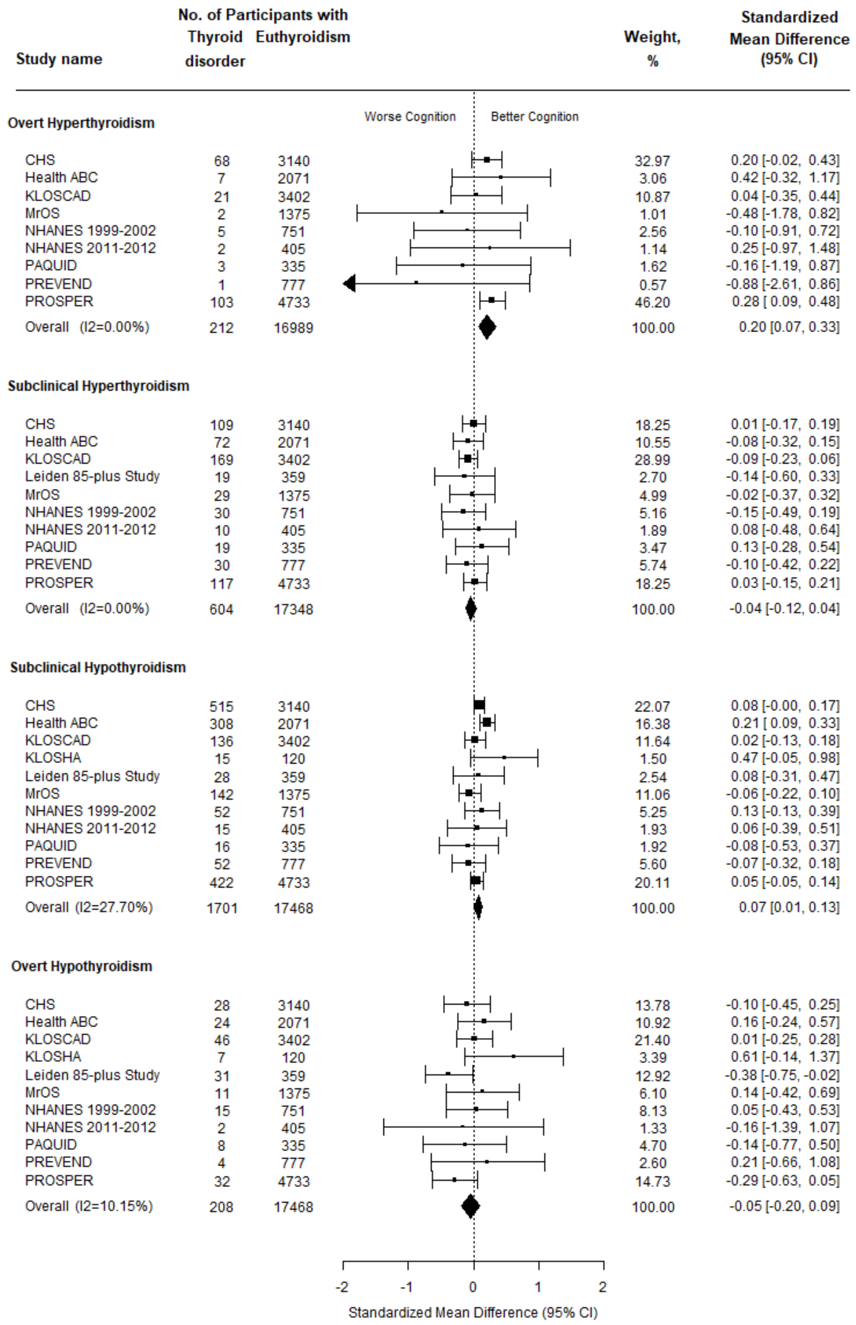

eFigure 4. Cross-sectional association between Thyroid Dysfunction and Executive Function

Standardized mean differences (SMDs) were adjusted for age and sex. Error bars indicate 95% confidence intervals. Abbreviated study names: CHS, Cardiovascular Health Study; Health ABC, Health, Aging and Body Composition Study; Korean Longitudinal Study on Cognitive Aging and Dementia; KLOSHA, Korean Longitudinal Study on Health and Aging; MrOS, Osteoporotic Fractures in Men Study; NHANES, National Health and Nutrition Examination Survey; PAQUID study, Personnes-Agées QUID study; PREVEND, Prevention of Renal and Vascular End-stage Disease Study; PROSPER, Prospective Study of Pravastatin in the Elderly at Risk.

© 2021 van Vliet NA et al. *JAMA Internal Medicine.*

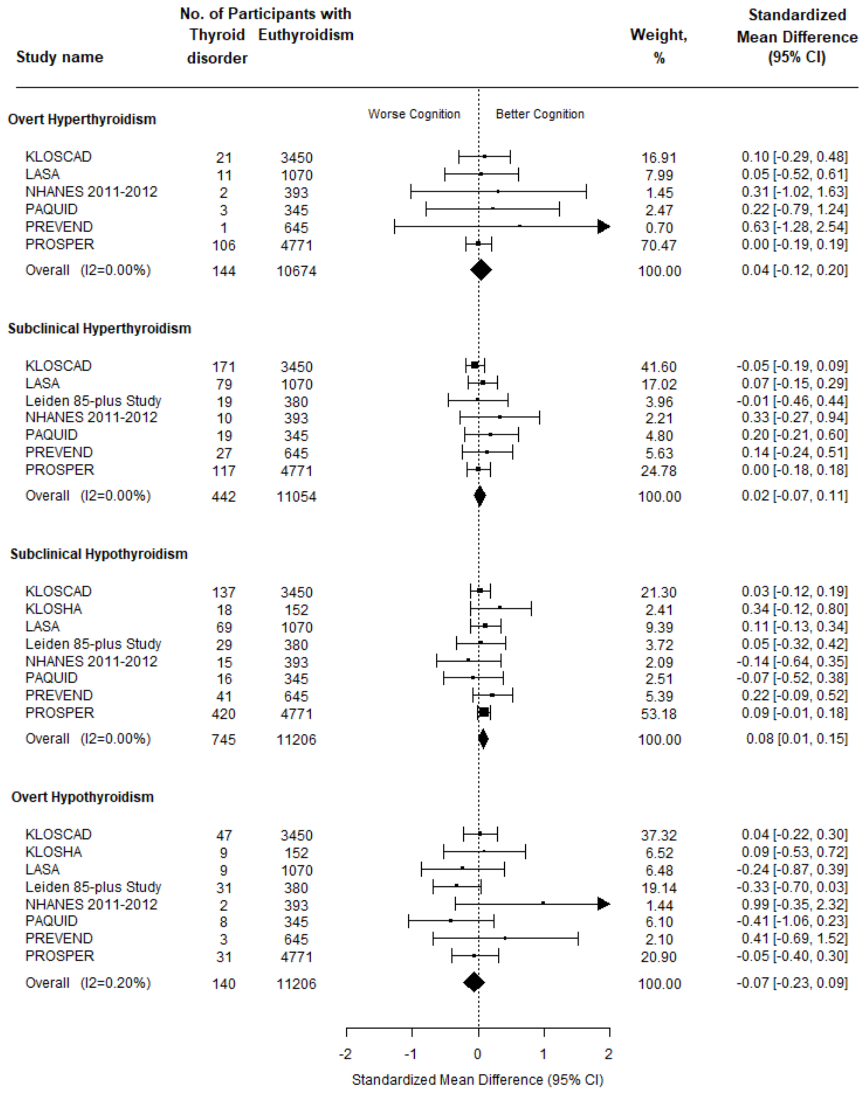

eFigure 5. Cross-sectional association between Thyroid Dysfunction and Memory

Standardized mean differences (SMDs) were adjusted for age and sex. Error bars indicate 95% confidence intervals. Abbreviated study names: KLOSCAD, Korean Longitudinal Study on Cognitive Aging and Dementia; KLOSHA, Korean Longitudinal Study on Health and Aging; LASA, Longitudinal Aging Study Amsterdam; LLS, Leiden Longevity Study; MMC, Mexican Memory Clinic; MrOS, Osteoporotic Fractures in Men Study; NHANES, National Health and Nutrition Examination Survey; PAQUID study, Personnes-Agées QUID study; PREVEND, Prevention of Renal and Vascular End-stage Disease Study; PROSPER, Prospective Study of Pravastatin in the Elderly at Risk.

© 2021 van Vliet NA et al. *JAMA Internal Medicine.*

Study name	Thyroid disorder Cases	Thyroid disorder Controls	Euthyroidism Cases	Euthyroidism Controls	Weight %	Hazard ratio (95% CI)
**Overt Hyperthyroidism**						
HUNT	4	206	483	30735	43.34	0.94 [0.35, 2.51]
KLOSCAD	2	27	120	3369	22.99	3.52 [0.86, 14.34]
Rotterdam Study	3	10	293	1308	33.68	1.65 [0.53, 5.14]
Overall (I2=10.63%)	9	243	896	35412	100.00	1.54 [0.76, 3.10]
**Subclinical Hyperthyroidism**						
HIMS	6	31	432	2805	7.98	0.97 [0.43, 2.17]
HUNT	27	911	483	30735	30.11	1.62 [1.10, 2.39]
KLOSCAD	2	143	120	3369	2.72	0.35 [0.09, 1.44]
LASA	10	64	112	790	12.10	1.00 [0.52, 1.90]
Leiden 85-plus	3	17	50	344	3.91	1.21 [0.38, 3.89]
PAQUID	3	13	109	213	3.95	0.46 [0.14, 1.46]
RERF	8	38	182	1063	10.11	1.47 [0.72, 3.00]
Rotterdam Study	27	93	293	1308	29.11	1.32 [0.89, 1.95]
Overall (I2=6.35%)	86	1310	1781	40627	100.00	1.23 [0.98, 1.56]
**Subclinical Hypothyroidism**						
HIMS	39	212	432	2805	28.44	1.15 [0.83, 1.60]
HUNT	31	1278	483	30735	23.21	1.15 [0.80, 1.65]
KLOSCAD	8	191	120	3369	5.93	1.25 [0.61, 2.57]
LASA	11	52	112	790	7.99	1.19 [0.64, 2.22]
Leiden 85-plus	6	26	50	344	4.28	1.53 [0.66, 3.58]
PAQUID	6	8	109	213	4.51	1.11 [0.49, 2.54]
RERF	13	89	182	1063	9.35	0.62 [0.35, 1.10]
Rotterdam Study	22	85	293	1308	16.29	1.01 [0.65, 1.56]
Overall (I2=0.00%)	136	1941	1781	40627	100.00	1.08 [0.91, 1.29]
**Overt Hypothyroidism**						
HIMS	2	10	432	2805	10.73	1.70 [0.42, 6.82]
HUNT	2	236	483	30735	10.73	0.39 [0.10, 1.56]
KLOSCAD	2	49	120	3369	10.63	0.93 [0.23, 3.76]
Leiden 85-plus	5	30	50	344	21.09	1.05 [0.42, 2.64]
PAQUID	1	3	109	213	5.62	1.25 [0.17, 9.07]
RERF	4	75	182	1063	18.83	0.31 [0.12, 0.84]
Rotterdam Study	5	19	293	1308	22.37	1.03 [0.43, 2.51]
Overall (I2=16.47%)	21	422	1669	39837	100.00	0.79 [0.48, 1.28]
RE Model						

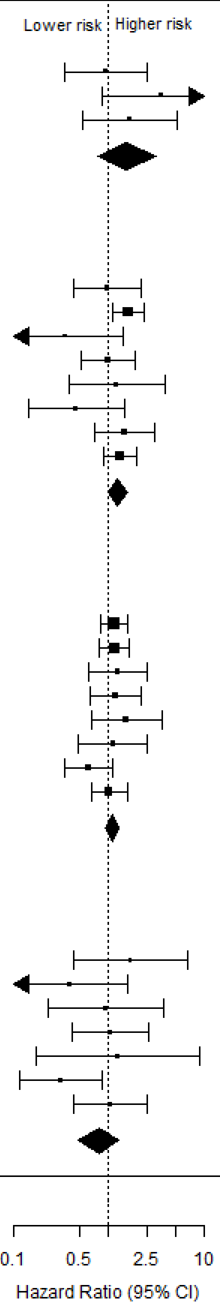

eFigure 6. Longitudinal Association between Thyroid Dysfunction and Incident Dementia

Hazard Ratios (HRs) were adjusted for age and sex. Error bars indicate 95% confidence intervals. Abbreviated study names: HIMS, Health in Men Study; HUNT, Trøndelag Health Study; KLOSCAD, Korean Longitudinal Study on Cognitive Aging and Dementia; LASA, Longitudinal Aging Study Amsterdam; PAQUID study, Personnes-Agées QUID study; RERF, Radiation Effects Research Foundation.

© 2021 van Vliet NA et al. *JAMA Internal Medicine.*
